# Continuity of Early Intervention Services in New York City During the COVID-19 Pandemic

**DOI:** 10.5195/ijt.2023.6553

**Published:** 2023-05-11

**Authors:** Stella Kasamba, Katharine H. McVeigh, Aurora Moraes, Ying Huang, Nora Puffett, Lidiya Lednyak

**Affiliations:** 1 Bureau of Early Intervention, New York City Department of Health and Mental Hygiene, New York City, New York, USA

**Keywords:** Children with disabilities, COVID-19, Early intervention services, Health services research, Teletherapy

## Abstract

In response to COVID-19, the New York City Early Intervention (EI) Program rapidly transitioned from in-person to teletherapy services. We describe the timing of service resumption among children who received EI services between March 1 and March 17, 2020. The proportion of children who transitioned to teletherapy-only was 25% as of March 24, rising to 78% by July 6. By December 31, 2020, 87% of the cohort had resumed either teletherapy or in-person services. Child age, race, language, and neighborhood poverty all predicted service resumption timing. Children with a diagnosis of autism spectrum disorder were more likely to transition to teletherapy, and children with only 1-2 domains of delay were more likely to discontinue services altogether. Continuity of EI services during the COVID-19 public health emergency was a critical priority. Timely policy changes facilitated swift return to services and avoided exacerbation of the long-standing racial disparities in access to EI services.

As the COVID-19 pandemic quickly spread across the United States in March 2020, it forced statewide shutdowns (Centers for Disease Control [CDC], 2022), including early childhood learning centers and schools, consequently disrupting the environments where children grow, learn, and develop, especially those with developmental disabilities (Egan et al., 2021). On March 13, 2020, in response to the need to limit community spread of COVID-19 while ensuring continuity of care for children and families, the New York City Department of Health and Mental Hygiene's Bureau of Early Intervention (BEI) issued guidance describing how, effective March 16, 2020, all New York City (NYC) Individualized Family Service Plan (IFSP) meetings would be conducted via conference call. Additional guidance was issued on March 18 pertaining to the use of teletherapy approaches to conduct child evaluations and deliver services. As per the Governor's “New York State on Pause” executive order, effective Tuesday, March 24, 2020, Early Intervention (EI) Services in NYC could only be provided in a limited set of approved childcare settings or through teletherapy until the pause was lifted. BEI and EI provider agencies and interventionists worked to put new procedures in place and rapidly transition families from in-person to teletherapy services.

EI is a federally mandated program administered by states and localities that offers services, at no cost to families, to children ages birth to three who have eligible developmental delays and disabilities. These services may include physical therapy, occupational therapy, speech therapy and other services based on the child's needs. (Centers for Disease Control and Prevention [CDC], 2019). EI services can help children with disabilities or developmental delays to acquire vital competencies that will improve their long-term academic achievement, behavior, and social skills. Research has shown that the brain develops rapidly in the first few years of life, and for this reason EI services are most beneficial when disabilities are identified early and addressed promptly (The National Early Childhood Technical Center [NECTAC], 2011). The positive effects of receiving EI services on the developmental trajectories of children with autism are also well documented (Klintwall et al., 2015). Delaying or skipping services has been associated with the need for more costly interventions later in life (Litt et al., 2018). For these reasons, it was crucial for the BEI to maintain access to EI services via teletherapy throughout the COVID-19 pandemic when social distancing policies prohibited or discouraged the delivery of services in-person.

Assessments and delivery of therapeutic services to young children with disabilities through teletherapy incorporated the use of video conferencing. Because of this, therapists and families were able to communicate using both speech and gestures despite being physically separated. While previous research has shown that the use of teletherapy to provide EI services can sometimes limit engagement and interaction with families, and that technical difficulties including poor internet connection, audio, and video problems can make it difficult for some families to use, EI teletherapy has also been found to be a highly effective and a practical solution when in-person services cannot be offered (Cason, 2009, 2011).

Monitoring the continuation of early intervention services during the pandemic was essential to ensure that children with developmental delays or disabilities received the support they needed to reach their full potential, and to identify whether the pandemic was exacerbating historic inequities in access to EI services. This report describes the resumption of EI services beginning on March 24, 2020, and identifies child characteristics associated with teletherapy uptake, delayed resumption of EI services until in-person services again became available, and discontinuation of services altogether.

## Methods

Data Source: Data were obtained from the New York Early Intervention System (NYEIS), the administrative data system used by the New York City Early Intervention Program.

Population: The study population included 15,205 children who were active in the NYC EI Program and who received one or more of the following therapeutic and support services between March 1 and March 17, 2020: Occupational Therapy (OT), Physical Therapy (PT), Speech and Language Therapy (ST) or Special Instruction (SI).

Dependent variables: Time of service resumption defined in continuous days, and EI service resumption status defined as(1) teletherapy uptake (resumed services during the teletherapy-only period from March 24 through July 5), (2) delayed resumption of services (resumed services after in-person services again became available, from July 6 through December 31, 2020) and (3) discontinuation of services (children who aged out of the EI program or did not resume services by December 31, 2020).

Independent variables: Child age on March 18, 2020 (< 12 months, 12-23 months, > 23 months), sex (male, female), language spoken at home (English, Spanish, Chinese, Hebrew/Yiddish, Russian, other), race/ethnicity (Asian/Pacific Islander/Native American, Black or African American, Latinx, White), neighborhood poverty (percentage of individuals living below the federal poverty level: < 10%, 10%–29%, 30%–100%), borough of residence (Bronx, Brooklyn, Manhattan, Queens, Staten Island), months of in-person services (0–3, 4–7, 8–11, 12+ months), whether the child received each of the following types of services during the period March 1–17, 2020: OT, PT, ST, and SI, and EI eligibility category (automatically eligible, diagnosed with autism spectrum disorder (ASD), diagnosed with developmental delays in one or two domains, diagnosed with developmental delays in 3-5 domains). Children who are determined to be eligible for EI services must be younger than three years old and have a documented disability or developmental delay in at least one of the following areas/domains: physical, cognitive, communicative, social-emotional, and adaptive.

Statistical Analysis: Cumulative frequency analyses were used to describe patterns of service re-uptake over time.

Frequency analysis and chi-square statistics were used to describe the study cohort and quantify the percentage of children falling into each EI service resumption category. Multinomial logistic regression with manual backward elimination was used to identify child characteristics that were independently associated with the timing of EI service resumption or discontinuation.

IRB: This work was carried out as public health activity to understand the impact of the COVID-19 public health emergency on EI Program operations. The Department of Health and Mental Hygiene IRB classified the work as exempt.

## Results

**Figure 1.**
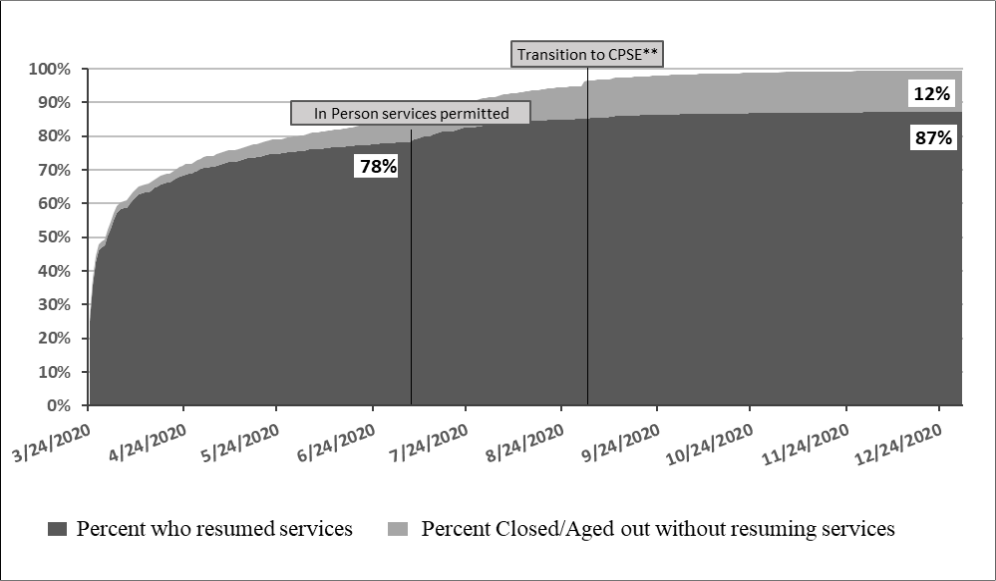
Resumption of NYC EI Services, March 24 through December 31, 2020

Note. This graph does not include the <1% of children who had neither aged out or resumed services as of 12/31/2020. **CPSE stands for Committee on Preschool Special Education. When children are 3 years of age, those determined eligible can transition from EI to early childhood special education services provided by CPSE. Children born from January through August typically transition in September, and those born from September through December typically transition in January.

Figure 1 displays the resumption of EI services between March 24 and December 31, 2020, as well as the discontinuation of services for children who didn't resume services before aging out. By March 24, 25% of children in the cohort had already transitioned to teletherapy and that percentage increased two-fold by March 30. By June 5, teletherapy uptake had increased to 76% of the cohort and it reached 78% by July 6 when the ban on the delivery of in-person services was lifted. Between July 7 and October 1, the proportion of children who had resumed services climbed to 87% where it stayed through December 31, 2020. Among the children who did not resume services, 7% aged out or formally withdrew from services during the teletherapy-only period, while another 5% did so by the end of the year. As of December 31, 2020, about 0.5% of children in the cohort were still within the eligible age range but had not resumed services.

Table 1 describes the characteristics of the study cohort and the distribution of service resumption for children with each characteristic. Children in the cohort, who were active in the NYC EI Program and received one or more OT, PT, ST or SI service between March 1 and March 17, 2020, were predominantly 24 months of age or older (75%), male (65%) and spoke English at home (64%). Other common home languages included Spanish (15%), Russian (7%), Yiddish or Hebrew (4%), and Chinese (Mandarin or Cantonese, 3%). The children in the cohort were disproportionately White (40%), while Black children were significantly under-represented (15%) relative to the NYC population of children under three years of age. Latinx and Asian children made up 33% and 12% of the cohort, respectively. Most children (65%) resided in moderate- to high-poverty neighborhoods and 16% in very high poverty neighborhoods. The distribution of children across the City's five boroughs resembled that of young children in NYC overall, but with slightly more hailing from Queens and fewer from Manhattan. A little over one-quarter (29%) of children were classified as having 1-2 domains of delay, 32% with 3-5 domains of delay, 30% with autism spectrum disorder (ASD), and 9% with a condition that confers automatic eligibility for EI services. Most children in the study cohort (71%) received speech and language therapy in-person before the pandemic and 59% received special instruction, while 42% and 38% received occupational therapy, and physical therapy respectively. Characteristics of the study cohort stratified by timing of resumption of EI therapeutic and support services can be found in Table 1b.

**Table 1.** Cohort* Characteristics Stratified by Timing of Resumption of General Services

	1b. Timing of Resumption of General Services					
	1a. Entire Cohort* N = 15,205		Before July 6, 2020 N = 11,886	July 6 - December 31, 2020 N = 1,374	Aged Out/Did not Resume Services N = 1,945	
	N	%	%	%	%	p
**Child's Age on March 18, 2020**						<0.001
< 12 months	751	5%	5%	9%	4%	
12-23 Months	3,030	20%	19%	36%	15%	
24-36 Months	11,424	75%	76%	55%	81%	
**Sex**						<0.001
Male	9,877	65%	65%	60%	66%	
Female	5,328	35%	35%	40%	34%	
**Language at Home**						<0.001
English	9,657	64%	66%	52%	55%	
Spanish	2,239	15%	15%	10%	15%	
Chinese (Mandarin, and Cantonese)	487	3%	3%	3%	5%	
Yiddish/Hebrew	679	4%	2%	19%	8%	
Russian	1,078	7%	7%	9%	8%	
Other	1,065	7%	7%	7%	9%	
**Race/Ethnicity**						<0.001
White	6,111	40%	38%	57%	40%	
Black	2,250	15%	16%	9%	14%	
Latinx	4,954	33%	34%	22%	30%	
Asian	1,890	12%	12%	12%	15%	
**ZIP Code Poverty Level**						<0.001
Low Poverty (0 to <10%)	2,743	18%	19%	14%	16%	
Moderate to High Poverty (10 to < 30%)	9,924	65%	65%	64%	67%	
Very High Poverty (30 to 100%)	2,499	16%	16%	23%	17%	
**Borough**						<0.001
Bronx	2,846	19%	20%	13%	15%	
Brooklyn	5,494	36%	34%	50%	40%	
Manhattan	1,624	11%	11%	6%	9%	
Queens	4,280	28%	28%	25%	30%	
Staten Island	961	6%	7%	6%	5%	
**Eligibility Category**						<0.001
Automatically Eligible	1,380	9%	10%	10%	5%	
ASD	4,532	30%	33%	21%	17%	
1-2 Domains of Delay	4,383	29%	26%	33%	44%	
3-5 Domains of Delay	4,910	32%	32%	36%	33%	
**Months of In-Person Services**						<0.001
0-3 Months	3,660	24%	23%	32%	24%	
4-7 Months	3,879	26%	25%	26%	26%	
8-11 Months	2,912	19%	20%	14%	19%	
12+Months	4,754	31%	32%	28%	31%	
**Receive Occupational Therapy (OT) In March, 2020**						<0.001
Yes	6,356	42%	45%	37%	26%	
No	8,849	58%	55%	63%	74%	
**Receive Physical Therapy (PT) In March, 2020**						<0.001
Yes	5,737	38%	38%	50%	30%	
No	9,468	62%	62%	50%	70%	
**Receive Speech and Language Therapy (ST) In March, 2020**						<0.001
Yes	10,774	71%	74%	56%	63%	
No	4,431	29%	26%	44%	37%	
**Receive Special Instruction (SI) In March, 2020**						<0.001
Yes	9,041	59%	63%	46%	48%	
No	6,164	41%	37%	54%	52%	

*Note*. *Active in March 2020 and received 1 or more OT, PT, ST or SI service between March 1 and March 17, 2020

Table 2 illustrates the timing of resumption of general services. As previously mentioned, 78% of children in the cohort resumed services by July 5, 2020 during the teletherapy-only period and another 9% resumed services after in-person services became available again on July 6, 2020. Children with the lowest percentage of service resumption during the teletherapy-only period included those whose home language is Yiddish or Hebrew (40%), who were not authorized for ST (70%), or SI (72%), or who had delays in only one or two domains (70%). Uptake of teletherapy-only services was highest among children who received OT (84%), ST (81%) and SI (83%), as well as among children diagnosed with autism spectrum disorder (ASD) (86%) or a condition that confers automatic eligibility (82%). Teletherapy-only uptake was higher among children living in the Bronx (84%), Manhattan (83%) or Staten Island (81%) than among those living in Queens (78%) or Brooklyn (73%) and was also higher among children who are Latinx (82%) and Black (82%) than among those who are Asian (75%) and White (75%).

During the period from July 6, 2020, through December 31, 2020, teletherapy continued to be the primary and preferred mode of EI service delivery, but in-person services were also permitted. During this period, another 9% of children in the cohort resumed services, presumably in response to the return of in-person services. These children were classified as having delayed resumption of services. Delayed resumption of services was most common among children whose home language was Yiddish or Hebrew (38%) or Russian (11%), who were younger than 12 months (16%) or 12-23 months (16%) of age on March 18, 2020, who were White (13%), lived in Brooklyn (12%) or in very high poverty neighborhoods (12%), received PT in-person in March 2020 (12%), had received in-person services for three or fewer months before the teletherapy-only period (12%), and also among those who did not receive ST (14%) or SI (12%) in-person before the switch to teletherapy. Delayed resumption of services was uncommon among children with addresses in Manhattan (5%).

Almost 13% of children in the cohort didn 't return to receiving services before December 31, 2020. Discontinuation of EI services was associated with lesser severity of need and language spoken at home. Discontinuation was higher among children with delays in only 1-2 domains (20%) and among children who did not receive OT (16%), SI (16%) or ST (16%). Discontinuation of services was also higher among children who spoke Yiddish or Hebrew (23%), Chinese (20%), or a less commonly spoken language at home. Discontinuation of services was least common among children diagnosed with ASD (8%) or with a condition that confers automatic eligibility for EI (7%).

**Table 2.** Timing of Resumption of General Services Stratified by Cohort* Characteristics

	Timing of Resumption of General Services			
	Before July 6, 2020	July 6 - December 31, 2020	Aged Out/Did not Resume Services	
	N = 11,886	N = 1,945	Services N = 1,374	
	%	%	%	p
Percent of Entire Cohort*	78%	9%	13%	
**Child's Age on March 18, 2020**				<0.001
< 12 months	75%	16%	9%	
12-23 Months	74%	16%	10%	
24-36 Months	80%	7%	14%	
**Sex**				<0.001
Male	79%	8%	13%	
Female	77%	10%	13%	
**Language at Home**				<0.001
English	81%	7%	11%	
Spanish	81%	6%	13%	
Chinese (Mandarin, and Cantonese)	72%	8%	20%	
Yiddish/Hebrew	40%	38%	23%	
Russian	75%	11%	14%	
Other	74%	9%	17%	
**Race/Ethnicity**				<0.001
White	75%	13%	13%	
Black	82%	6%	12%	
Latinx	82%	6%	12%	
Asian	75%	9%	16%	
**ZIP Code Poverty Level Group**				<0.001
Low Poverty (0 to <10%)	82%	7%	11%	
Moderate to High Poverty (10 to < 30%)	78%	9%	13%	
Very High Poverty (30 to 100%)	75%	12%	13%	
**Borough**				<0.001
Bronx	84%	6%	10%	
Brooklyn	73%	12%	14%	
Manhattan	83%	5%	11%	
Queens	78%	8%	14%	
Staten Island	81%	8%	11%	
**Eligibility Category**				<0.001
Automatically Eligible	82%	10%	7%	
ASD	86%	6%	8%	
1–2 Domains of Delay	70%	10%	20%	
3–5 Domains of Delay	77%	10%	13%	
**Months of receiving In-person Services**				<0.001
0–3 Months	75%	12%	13%	
4–7 Months	78%	9%	13%	
8–11 Months	81%	7%	13%	
12+Months	79%	8%	13%	
**Receive Occupational Therapy (OT) In March, 2020**				<0.001
Yes	84%	8%	8%	
No	74%	10%	16%	
**Receive Physical Therapy (PT) In March, 2020**				<0.001
Yes	78%	12%	10%	
No	78%	7%	14%	
**Receive Speech and Language Therapy (ST) In March, 2020**				<0.001
Yes	81%	7%	11%	
No	70%	14%	16%	
**Receive Special Instruction (SI) In March, 2020**				<0.001
Yes	83%	7%	10%	
No	72%	12%	16%	

*Note*. *Active in March 2020 and received one or more OT, PT, SP or SI service between March 1 and March 17, 2020

Table 3 presents results of the multinomial logistic regression analysis. Factors associated with higher odds of delayed resumption of EI services, in comparison to timely resumption of services (i.e., before July 6, 2020), included age 12-23 months on March 18, 2020, living in a moderate to very high poverty neighborhood, residing in Brooklyn, Queens or Staten Island and having 3-5 domains of delay. Children who had only received in-person services in March 2020 for three months or less were also more likely to delay resumption of services compared to those who received in-person services for at least 12 months (OR= 1.4; 95% CI= 1.2 to 1.7; p = 0.001). Additionally, White children who predominantly spoke a language other than English at home were more likely to delay resumption of EI services compared to White children whose dominant language is English (OR= 2.3; 95% CI= 2.0 to 2.8; p <.001). Characteristics associated with higher odds of discontinuation of services in comparison to timely resumption of services included being 24 months or older on March 18, 2020, being identified as Black or Asian and speaking English at home, residing in a moderate to very high poverty neighborhood, living in Queens, having 1-2 domains of delay, and having received in-person services before the teletherapy period for at least 12 months. White children whose dominant language was not English were also more likely to have discontinued services (OR= 2.1; 95% CI= 1.7 to 2.4; p <.001) compared to White children who speak English at home.

**Table 3.** Characteristics Associated with Delayed Resumption or Discontinuation of EI Services in New York City During the COVID-19 Public Health Emergency

	Delayed Resumption (Services After July 6, 2020)				Discontinuation (No Services by December 31, 2020)			
	OR	LCL	UCL	p	OR	LCL	UCL	p
**Child's Age on March 18, 2020**								
< 12 months	1.1	0.9	1.5	0.442	0.5	0.4	0.6	<0.001
12-23 Months	1.8	1.5	2.1	<0.001	0.6	0.5	0.7	<0.001
24-36 Months*	Ref	–	–		Ref	–	–	
**Home Language and Race**								
White - English*	Ref	–	–		Ref	–	–	
Black or African American - English	0.6	0.5	0.7	<0.001	1.2	1.0	1.5	0.022
Latinx - English	0.7	0.5	0.8	<0.001	1.2	1.0	1.4	0.084
Asian/PI/NA - English	1.0	0.7	1.3	0.788	1.4	1.1	1.8	0.009
White - Other than English	2.3	2.0	2.8	<0.001	2.1	1.7	2.4	<0.001
Black or African American - Other than English	0.4	0.2	0.7	0.001	0.8	0.5	1.2	0.284
Latinx - Other than English	0.4	0.3	0.5	<0.001	0.6	0.5	0.8	<0.001
Asian/PI/NA Other than English	0.6	0.5	0.8	<0.001	1.0	0.8	1.2	0.635
ZIP Code Poverty Level Group								
Very High Poverty (30 to 100%)	2.8	2.2	3.5	<0.001	1.6	1.3	1.9	<0.001
Moderate to High Poverty (10 to< 30%)	1.4	1.1	1.6	0.002	1.2	1.0	1.4	0.028
Low Poverty (0 to <10%)	Ref	–	–		Ref	–	–	
**Borough**								
Bronx	1.0	0.7	1.3	0.953	0.8	0.6	1.0	0.026
Brooklyn	1.7	1.3	2.2	<0.001	1.2	1.0	1.4	0.140
Queens	1.6	1.2	2.0	0.001	1.4	1.1	1.7	0.001
Staten Island	1.6	1.2	2.3	0.005	1.2	0.9	1.6	0.207
Manhattan	Ref	–	–		Ref	–	–	
**Eligibility Category**								
Automatically Eligible	1.2	0.9	1.5	0.180	0.48	0.4	0.6	<0.001
ASD	0.9	0.8	1.1	0.287	0.46	0.4	0.5	<0.001
3–5 Domains of Delays	1.2	1.1	1.5	0.008	0.87	0.8	1.0	0.036
1–2 Domains of Delays	Ref	–	–		Ref	–	–	
**Months of In-person Services**								
0–3 Months	1.4	1.2	1.7	0.001	0.8	0.7	0.9	0.003
4–7 Months	1.1	0.9	1.3	0.285	0.8	0.7	1.0	0.019
8–11 Months	0.8	0.6	0.9	0.003	0.8	0.7	1.0	0.017
12+Months	Ref	–	–		Ref	–	–	
Receive Occupational Therapy (OT)*								<0.001
Yes	0.9	0.8	1.0	0.032	0.6	0.5	0.7	
No	Ref	–	–		Ref	–	–	
Receive Physical Therapy (PT)*								
Yes	1.0	0.9	1.2	0.890	0.5	0.4	0.6	<0.001
No	Ref	–	–		Ref	–	–	
Receive Speech and Language Therapy (ST)*								
Yes	0.7	0.6	0.8	<0.001	0.5	0.4	0.5	<0.001
No	Ref	–	–		Ref	–	–	
Receive Special Instruction (SI)*								
Yes	0.7	0.6	0.8	<0.001	0.5	0.5	0.6	<0.001
No	Ref	–	–		Ref	–	–	

*Note.* * Received these services between March 1 and March 17, 2020

## Discussion

The NYC EI Program rebounded quickly from the suspension of in-person services on March 24, 2020. Within a week of the suspension date, 50% of children had transitioned to teletherapy and by July 5, 2020, the end of the teletherapy-only period, 78% of children who had been receiving EI services in-person prior to March 24, 2020, were receiving telehealth services. The return of in-person services on July 6, 2020, resulted in an additional 9% of children resuming EI services. These children were more often younger than 24 months at the time in-person services were suspended, from households where the dominant language was a language other than English, especially if they were White, from poorer neighborhoods, and less likely to have a diagnosis of ASD compared to the children who resumed services more quickly. The remaining 13% of children never resumed services. About half of these children aged out of the Program during the teletherapy-only period, while all but 0.5% aged out by the beginning of the 2020–21 school year. In addition to being older than children who quickly transitioned to teletherapy, children who discontinued services were more likely to be both White and from households that speak a language other than English, to live in neighborhoods with higher levels of poverty, and much more likely to have only one or two domains of delay.

Continuity of EI services during the COVID-19 public health emergency was a critical priority of the NYC Bureau of Early Intervention (BEI). The Bureau had concerns about whether and how quickly families would be able to resume services and whether racial disparities in access to services would be exacerbated. In response, the BEI immediately implemented a multifaceted approach to keep families in care including issuing detailed policy guidance to the provider community, updating the NYC EIP website and Text-to-Families messaging to inform families that services were being delivered remotely and the benefits of teletherapy, modifying EI policy to permit a pause in services during the teletherapy-only period, adjusting service plans in response to changing family situations, and phoning families to facilitate their transition to teletherapy (New York State Department of Health, 2020).

Rapid resumption of EI services was critical in mitigating the potential damage that may have been caused by the pandemic on children's development. This study is particularly significant considering the COVID-related developmental and learning loss that has been recently documented. A study of children under three years of age by Deoni et al. (2021) comparing cognitive scores in years 2021 and 2020 to years 2011 to 2019 indicated that children born during the pandemic had poorer cognitive, motor, and verbal performance scores compared to those born before the pandemic. The National Center for Education Statistics also examined patterns in the reading and math scores of 9-year-old students to assess pandemic-related change. They found that the typical math and reading scores fell by five and seven points, respectively, in 2022. This was the first ever decrease in mathematics and the greatest reading mean score drop since 1990 (The Nation's Report Card, 2022).

We are heartened to know that most children in our care transitioned quickly to teletherapy and continued to receive services throughout the emergency, and that pre-existing racial inequities in access to EI services were not exacerbated.

Additionally, children with more intensive service needs were less likely to discontinue services and those with ASD were also less likely to delay resumption. Our findings are consistent with research from previous studies that indicated widespread acceptance and success with telehealth, particularly among children with ASD and among those with other developmental disabilities (Little et al., 2018; Pickard et al., 2016). The successful transition to teletherapy demonstrates that telehealth is a viable method of EI service provision that may be especially beneficial for families that have difficulty accessing in-person services.

Lower rates of teletherapy uptake among children who speak languages other than English at home are not surprising since any communication challenges stemming from differing levels of fluency in a common language could be exacerbated in a virtual setting. This is supported by a recent telemedicine study by Payán et al. (2022), which found that patients who did not speak English well had difficulty setting up and using cloud-based video conferencing apps like Zoom for their medical appointments (Payán et al., 2022). Language is also tied to culture and some cultures are less receptive to video calls than others. Among Asian children, lower rates of teletherapy uptake reflect historically low rates of EI referrals among Asian children with less severe delays. A study investigating patient characteristics related to telemedicine use during the COVID-19 pandemic found that being Asian and preferring a language other than English were both independently linked to fewer telehealth visits (Elberly et al., 2020).

We were not able to measure access to technology as a barrier to service continuity, however, the strong negative association between neighborhood poverty and teletherapy uptake is aligned with findings from Cole et al., (2019) that children in low-income neighborhoods had less access to EI teletherapy because of inadequate internet connectivity and dependability. At the same time, however, teletherapy may have improved EI access for families from low-income neighborhoods by removing barriers related to therapist transportation/parking and scheduling (Ellison et al., 2021; Sutherland et al., 2019).

A limitation of this study is our inability to determine which services delivered after July 6, 2020 were delivered in-person. We assume that children with delayed resumption of services were waiting for the return of in-person services, but we have no way of determining whether they had a mix of teletherapy and in-person services or only in-person services. We also don't know how many children who participated in teletherapy-only services before July 6, 2020 subsequently shifted to some or all in-person services thereafter. Furthermore, this study does not describe how children's service plans may have been modified in response to the challenges of the remote environment, although we know from our administrative data that overall and controlling for age, there was only a slight dip in the average number of services received per child per day from March to June 2020, followed by an increase from July 2020 through June 2021, and stabilization at or near pre-COVID-19 levels from July 2021 through June 2022.

Timely policy changes facilitated swift return to services and avoided exacerbation of the long-standing racial disparities in access to EI services. The robust uptake of teletherapy and continuity of EI services throughout the COVID-19 public health emergency suggests that much of the potential harm caused by the suspension of in-person services may have been mitigated. Most children missed only 1-2 weeks of service and half of those who discontinued services aged out within three months. Additional research is needed to better understand the high discontinuation of EI services among Asian children, who are also the racial/ethnic group with the lowest 2022 EI referral rate. Further investigation into how the composition and quantity of EI services received changed from 2019 through 2021 is also warranted. If teletherapy continues to be used for EI evaluations going forward, research validating evaluation tools administered remotely will be essential to ensure children are not inappropriately included in or excluded from services. Additionally, better information is needed about the effectiveness of EI teletherapy overall, for different types of therapies and for children with different needs so that best practices can be identified, documented, and applied. Finally, longitudinal studies are needed to ascertain the long-term impact of the COVID pandemic on child development.

The COVID-19 pandemic presented some challenges for the delivery of EI services to young children and their families. However, teletherapy emerged as a valuable tool for providing continuity of services during the pandemic. Even though the uptake of teletherapy presented some challenges, especially among the non-English speaking families, we take comfort in knowing that most children and their families continued to meet regularly with their interventionists and work toward their developmental goals, and that historical inequities in EI access were not exacerbated. Overall, teletherapy can be a highly effective tool for supporting young children's development, but its use needs to be carefully planned and implemented to ensure that it is effective and accessible for all families that want it. With the right strategies in place, teletherapy can help ensure that children continue to receive the support they need to reach their full potential, even during a public health emergency.
